# Genetic Susceptibility to Chronic Kidney Disease – Some More Pieces for the Heritability Puzzle

**DOI:** 10.3389/fgene.2019.00453

**Published:** 2019-05-31

**Authors:** Marisa Cañadas-Garre, Kerry Anderson, Ruaidhri Cappa, Ryan Skelly, Laura Jane Smyth, Amy Jayne McKnight, Alexander Peter Maxwell

**Affiliations:** ^1^Epidemiology and Public Health Research Group, Centre for Public Health, Queen’s University of Belfast, Belfast, United Kingdom; ^2^Regional Nephrology Unit, Belfast City Hospital, Belfast, United Kingdom

**Keywords:** telomeres, copy number variants, single nucleotide polymorphisms, whole exome sequencing, mitochondria, chronic kidney disease

## Abstract

Chronic kidney disease (CKD) is a major global health problem with an increasing prevalence partly driven by aging population structure. Both genomic and environmental factors contribute to this complex heterogeneous disease. CKD heritability is estimated to be high (30–75%). Genome-wide association studies (GWAS) and GWAS meta-analyses have identified several genetic loci associated with CKD, including variants in *UMOD, SHROOM3*, solute carriers, and E3 ubiquitin ligases. However, these genetic markers do not account for all the susceptibility to CKD, and the causal pathways remain incompletely understood; other factors must be contributing to the missing heritability. Less investigated biological factors such as telomere length; mitochondrial proteins, encoded by nuclear genes or specific mitochondrial DNA (mtDNA) encoded genes; structural variants, such as copy number variants (CNVs), insertions, deletions, inversions and translocations are poorly covered and may explain some of the missing heritability. The sex chromosomes, often excluded from GWAS studies, may also help explain gender imbalances in CKD. In this review, we outline recent findings on molecular biomarkers for CKD (telomeres, CNVs, mtDNA variants, sex chromosomes) that typically have received less attention than gene polymorphisms. Shorter telomere length has been associated with renal dysfunction and CKD progression, however, most publications report small numbers of subjects with conflicting findings. CNVs have been linked to congenital anomalies of the kidney and urinary tract, posterior urethral valves, nephronophthisis and immunoglobulin A nephropathy. Information on mtDNA biomarkers for CKD comes primarily from case reports, therefore the data are scarce and diverse. The most consistent finding is the A3243G mutation in the *MT-TL1* gene, mainly associated with focal segmental glomerulosclerosis. Only one GWAS has found associations between X-chromosome and renal function (rs12845465 and rs5987107). No loci in the Y-chromosome have reached genome-wide significance. In conclusion, despite the efforts to find the genetic basis of CKD, it remains challenging to explain all of the heritability with currently available methods and datasets. Although additional biomarkers have been investigated in less common suspects such as telomeres, CNVs, mtDNA and sex chromosomes, hidden heritability in CKD remains elusive, and more comprehensive approaches, particularly through the integration of multiple –“omics” data, are needed.

## Introduction

Chronic kidney disease is a major global health problem with an increasing prevalence (Levey et al., 2007; Bash et al., 2009; Centers for Disease Control and Prevention, 2015). By 2040, it is estimated that CKD will have become the fifth leading cause of death (Foreman et al., 2018). This increasing CKD burden is driven in part by aging population structure (CKD is ∼8x more common in adults > 70 years old compared to persons < 40 years of age) (Bash et al., 2009; Centers for Disease Control and Prevention, 2015). Diabetes and hypertension are common risk factors for kidney damage (Kazancioğlu, 2013) and are therefore major contributors to the increased CKD prevalence (Bash et al., 2009).

There is a marked gender imbalance in CKD with a higher incidence (11.0 vs. 9.6 per 1,000 person-years) and higher prevalence (16.0% vs. 12.4%) in women (Bash et al., 2009; Centers for Disease Control and Prevention, 2015). Nevertheless, women have a lower risk of CKD progression and men are more likely to develop ESRD (Ricardo et al., 2018).

Chronic kidney disease is a complex heterogeneous disease, with contributions from both genomic and environmental factors. CKD heritability has been estimated to be high (30–75%) (Satko and Freedman, 2005; O’Seaghdha and Fox, 2011; Regele et al., 2015). CKD can be identified by well-established clinical biomarkers such as SCr levels, eGFR, albuminuria, or UACR (Cañadas-Garre et al., 2018a,b). Unfortunately, these clinical biomarkers are limited in their utility to predict individual risk of CKD or likelihood for later progression to ESRD. Major efforts have been made to understand the heritability in CKD but the causal pathways remain incompletely understood. Four major approaches have been proposed to uncover the missing heritability; exploration of rare variants, increased samples sizes, study of molecular factors not involving variants in the DNA sequence and consideration of whether family studies overestimated heritability risk (Bourrat et al., 2017). In CKD, meta-analyses of GWAS have provided a useful and relatively inexpensive strategy to increase the statistical power by combining data summaries from different individual GWAS, helping to attenuate the issue of small sample size and identifying many genetic loci associated with CKD and/or kidney function traits (Köttgen et al., 2009, 2010; Chambers et al., 2010; Böger et al., 2011; Parsa et al., 2013; Pattaro et al., 2016; Gorski et al., 2017). Rare variants in *UMOD, SHROOM3*, solute carriers, and E3 ubiquitin ligases have also been associated with CKD, eGFR or SCr (Köttgen et al., 2012; Sveinbjornsson et al., 2014; Prokop et al., 2018). However, these genetic markers do not account for all the susceptibility to CKD, therefore other factors must be contributing to the missing heritability. Part of the missing heritability may correspond to genetic interactions (epistasis), rather than to missing variants (Zuk et al., 2012). Telomere length is a biological factor that has been associated with CKD prevalence and/or CKD progression in a small number of studies (Ameh et al., 2017). Structural variants, such as CNVs, insertions, deletions, inversions and translocations are, in general, poorly covered in commercial arrays and may explain part of the missing heritability (Manolio et al., 2009). Mitochondrial proteins, encoded by nuclear genes, and specific mtDNA encoded genes have also been associated with CKD (Skelly et al., 2019). The sex chromosomes, often excluded from GWAS studies, may help explain gender imbalances in CKD.

In this review, we outline some recent findings on molecular biomarkers for CKD (telomeres, CNVs, mtDNA variants, X and Y chromosomes) that typically have received less attention than single nucleotide polymorphisms (SNPs) present on, or imputed from, GWAS arrays. These less commonly studied biomarkers may be part of the “missing heritability” for CKD.

### Telomeres and CKD

**FIGURE 1:**
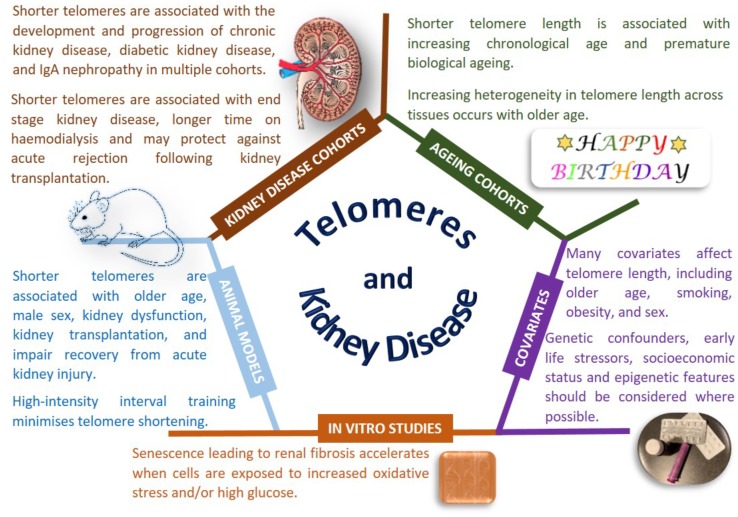
Telomeres and kidney disease. Copyright disclosure: mouse: https://commons.wikimedia.org/wiki/File:Vector_diagram_of_laboratory_mouse_%28black_and_white%29.svg; https://creativecommons.org/licenses/by-sa/4.0/deed.en; attribution, “By Gwilz [CC BY-SA 4.0 (https://creativecommons.org/licenses/by-sa/4.0)], from Wikimedia Commons. Kidney: https://commons.wikimedia.org/wiki/File:Kidney_Cross_Section.png; By Artwork by Holly Fischer [CC BY 3.0 (https://creativecommons.org/licenses/by/3.0)], via Wikimedia Commons.

Telomeres are specialized nucleoprotein complexes that help protect the ends of linear chromosomes (Sfeir, 2012). There are inter-individual and intra-individual differences in the length of telomeres. Shorter telomere length has been associated with multi-system diseases, early life stressors, increasing chronological age and all-cause mortality (Dlouha et al., 2014; De Meyer et al., 2018; Desai et al., 2018; Mangaonkar and Patnaik, 2018; Wang et al., 2018; Willis et al., 2018) (Figure 1). The majority of studies have analyzed relative telomere length in peripheral blood leukocytes, but telomere length differs between tissues within a single individual, with greater heterogeneity in telomere length evident in older people (Butler et al., 1998; Dlouha et al., 2014). Telomere length has a reported heritability of 28–82%, however, not all genetic factors (Broer et al., 2013; Codd et al., 2013) or environmental influences on telomere length are known (Cubiles et al., 2018; Dugdale and Richardson, 2018; Gao et al., 2018; Lu et al., 2018). Meta-analysis of telomere length may help confirm discovery associations across multiple collections, however, this is challenging with different wet-lab techniques (such as time at sample collection, storage and processing of biological material, absolute compared to relative telomere length evaluation, platform employed) and *in silico* analyses (such as normalization, controls, covariates, association, and correction tools) having significant effects on the reported measurements. There is also limited traditional epidemiological evidence exploring the mechanistic basis or causality of reported associations.

Nonetheless, there is evidence that telomere length is associated with disease states, particularly age-related diseases, beyond the most commonly studied cancers (Rizvi et al., 2014; Jafri et al., 2016). Conflicting reports have been published for the association of telomere length with renal disease, however, most publications, albeit in relatively small sample sizes with modest significance values, report that shorter telomere length is associated with renal dysfunction. Shorter telomeres have been reported as associated with progression of CKD (defined as a doubling of baseline SCr and/or ESRD), in the MMKD (*n* = 59 patients had confirmed CKD progression) and CRISIS (*n* = 105 patients had confirmed CKD progression) studies, with the effect size strengthened by smoking and the presence of diabetes (Raschenberger et al., 2015). Telomere shortening has been associated with IgAN in 177 patients, but not in 30 patients with DKD or 30 patients with FSGS compared to 83 controls (Lu et al., 2014). A study examining DNA from peripheral blood and urine in 15 patients with IgAN showed shorter telomere length correlated with declining renal function (Szeto et al., 2005). Multiple studies have been performed for DKD, with the majority linking shorter telomere length to the development and progression of kidney disease in people with both type 1 (Astrup et al., 2010, 273 patients; Fyhrquist et al., 2010, 176 patients, 21 progressed) and type 2 diabetes (Tentolouris et al., 2007, 168 patients; Verzola et al., 2008, 17 patients; Testa et al., 2011, 501 patients; Gurung et al., 2018, 691 patients). Shorter telomere length is associated with diabetic complications (Testa et al., 2011) and all-cause mortality (Astrup et al., 2010). The Heart and Soul Study is a longitudinal cohort of individuals with stable coronary heart disease; shorter telomere length at baseline and more rapid telomere shortening over 5 years were associated with reduced kidney function, but these changes were not significant when accounting for age (Bansal et al., 2012). It is noteworthy that the largest study published considered less than 1,000 individuals (Testa et al., 2011), which provides limited power to draw robust conclusions in this era of mega-consortia studying the genetics of CKD.

Premature telomere shortening is associated with duration of dialysis treatment in terms of months to years (Boxall et al., 2006). A cross-sectional study of 175 hemodialysis patients reported shorter telomere length in men with CKD, despite women having an older average age in this cohort; association of shorter telomeres was also observed with increasing age and male sex (Carrero et al., 2008). Shorter telomeres were associated with CKD in 203 Japanese hemodialysis patients compared to 203 age and sex-matched controls without CKD, with shorter telomeres also associated with new onset cardiovascular events (Hirashio et al., 2014). A less reactive immune system is associated with healthy aging in the general population and ESRD enhances premature immunological aging with shorter telomeres observed in 137 patients with ESRD compared to 144 individuals without kidney disease (Betjes et al., 2011).

Histologically normal and abnormal human kidney tissue samples from 24 individuals highlighted age-related shorter telomere length with telomeres typically shorter in the cortex than in the medulla (Melk et al., 2000). Premature senescence is an important feature of renal fibrosis that accelerates when cells are exposed to stressful environments such as more ROS and higher glucose (Verzola et al., 2008; Carracedo et al., 2013; Cao et al., 2018). Increasing age and sex related telomere shortening is observed in kidneys, with shorter telomeres observed in male rats (Cherif et al., 2003). Multiple animal models of kidney disease show telomere shortening associated with renal dysfunction, however, a careful experimental design is required for accurate telomere measurement (Hastings et al., 2004). Exploring renal ischemia/reperfusion injury in wild-type and telomerase deficient mice also suggests that shorter telomeres impair recovery from acute kidney injury (Westhoff et al., 2010; Song et al., 2011; Cheng et al., 2015). Severe renal failure induces telomere shortening (Wong et al., 2009) with rapid telomere loss observed during kidney transplantation in a rat model of chronic rejection (Joosten et al., 2003). Tucker and colleagues demonstrated that high-intensity interval training was beneficial protecting against telomere erosion in a rat model of CKD (Tucker et al., 2015).

Large-scale studies using carefully collected biological samples with harmonized phenotypes and analysis protocols will help determine the true association of telomere length for CKD. Potential therapies exist to minimize premature telomere shortening (Townsley et al., 2016; Rodrigues et al., 2017), but further work is needed to define the mechanistic links between telomere length and kidney function.

### Copy Number Variation and Larger Chromosomal Re-arrangements Association With CKD

Copy number variants are genetic structural variants which involve DNA regions being deleted or duplicated. This can occur throughout the genome affecting stretches of DNA ranging from kilo- to mega-base pairs in length and can result in abnormal gene amplification (Thapar and Cooper, 2013; Sampson, 2016). CNVs can be both inherited and arise *de novo*, and are increasingly being recognized as a significant source of genetic variation relating to both population diversity and disease, including renal diseases (Sampson, 2016), neuropsychiatric diseases (Lew et al., 2018), and cancer (Liang et al., 2016).

There is often uncertainty about the genetic basis of CKD in pediatric patients, but recent studies have indicated that chromosomal microarrays have the potential to partly address this. Verbitsky et al. (2015) assessed 419 children enrolled in the CKD in children (CKiD) study alongside 21,575 children and adults who had undergone microarray genotyping for non-CKD studies. CNV disorders were identified in 31 children with CKD and 10 known pathogenic genomic disorders were detected including *HNF1B* deletion at 17q12. A further 12 pathogenic genomic imbalances were identified using this technique, distributed evenly among patients diagnosed with congenital and non-congenital forms of CKD. Overall, large gene-altering CNVs were more common in the CKiD population compared with the controls (38 vs. 23%), but the specific genetic alterations identified in several of the individuals would require personalized recommendations in future healthcare.

Copy number variants have been linked to CAKUT (Sanna-Cherchi et al., 2012; Caruana et al., 2014; Bekheirnia et al., 2017; Siomou et al., 2017). In a study by Caruana et al. (2014), DNA from 178 Australian children who presented with any abnormality associated with CAKUT was screened using SNP arrays. In total, CNVs were identified in 18 children, of which 11 children presented with genomic disorders of unknown significance. Of these 11 participants, four were reported as having duplications of 1q23.1, 4p16.1, 7q33, and 8q13.2q13.3 regions, containing genes *NEPH1, SLC2A9, AKR1B1*, and *EYA1*, respectively. Each of these genes have previously been associated with renal abnormalities.

In an investigation undertaken by Siomou et al. (2017), seven children with CAKUT were assessed from three unrelated families using array comparative genomics hybridization. Of these participants, one reportedly had ureterovesical junction obstruction and a 1.4 Mb deletion at 17q12, containing two genes, *HNF1B*, which has been previously associated with CAKUT, and ACACA (Thomas et al., 2011; Caruana et al., 2014).

A recent study published by Bekheirnia et al. (2017), suggested whole exome sequencing (WES) as a viable method to detect CNVs in individuals with CAKUT. These investigators performed WES in 112 individuals from 62 families, to identify SNVs and CNVs in 35 genes previously related to CAKUT. They identified a *de novo* triplication in one family at 22q11, and overall, 6.5% of the individuals assessed in this investigation were shown to have pathogenic CNVs.

Posterior urethral valves are one of the most common causes of CKD in children. Faure et al. (2016) assessed the phenotypic effects of and relationship between renal outcomes and CNVs in 45 boys with PUV. In total, 13 CNVs were identified in 12 boys, two of which, at positions 3p25.1p25.2 and 17p12, were pathogenic in nature. Additionally, those CNVs identified which were > 100 kb in size, were significantly associated with earlier onset of renal failure in children with PUV.

Nephronophthisis (NPH) is a Mendelian genetic disease, which often leads to ESRD by around 13 years of age. Snoek et al. (2018) sought to investigate the prevalence of NPH in adult-onset ESRD, through assessment of the CNVs in the *NPHP1* gene (>90 kb) because a homozygous full gene deletion is a prominent cause of NPH. These investigators assessed 5,606 adult renal transplant recipients, 26 of whom showed evidence of the homozygous *NPHP1* deletion, compared to none of the 3,311 controls. Despite this, only 12% of the patients with the homozygous *NPHP1* gene deletion were clinically diagnosed with NPH.

Copy number variants have also been investigated in association with IgAN, which is the most common cause of primary glomerulonephritis (Ai et al., 2016). The multi-allelic CNV in the defensin alpha 1 and alpha 3 gene locus (*DEFA1A3*) was assessed in two independent IgAN cohorts of Chinese Han individuals (Ai et al., 2016). This locus can present as tandem repeats of a 19kb DNA stretch, containing one copy of either *DEFA1* or *DEFA3*, and several bi-allelic polymorphisms. The protein products of *DEFA1A3*, human neutrophil peptides 1–3, are abundant neutrophil granule proteins and function in the regulation of both the complement system and pro-inflammatory cytokine production. Each of these have been previously linked with IgAN.

Evaluation of the presence of CNVs yields potentially useful clinical information, especially for pediatric individuals with CKD.

Copy number variants in the human genome are likely to contribute to healthy development, but have additionally been linked to several human diseases (Sampson, 2016; Liang et al., 2016; Lew et al., 2018). The molecular mechanisms that trigger the formation of CNVs are not fully understood, but recurrent CNVs with common breakpoints reportedly arise through unequal meiotic or non-allelic homologous recombination (Arlt et al., 2012). Recent evidence has suggested that *de novo* and non-recurrent CNVs may develop following either replicative errors, chromosome shattering or chromothripsis (Kloosterman et al., 2011; Arlt et al., 2012; Nazaryan-Petersen et al., 2018).

Replication stress occurring during DNA replication has been linked to the collapse of the DNA replication fork and creation of a single-ended double strand break (Arlt et al., 2012). It has been considered that this could result in a high frequency of *de novo* CNVs. Both the fork collapse and strand break could result in the activation of damage checkpoint and repair pathways to correctly reactivate replication, thus preventing the creation of structural variants. However, CNVs are understood to be created if this reactivation occurs in an incorrect location using a template switch, or when an incorrect repair occurs, which joins two distant DNA breaks and causes a large deletion (Arlt et al., 2012). Any present mutations which inhibit the ability of the cell to accurately respond to a collapsed fork, are thought to ultimately increase the formation of CNVs (Arlt et al., 2012).

### Single Nucleotide Polymorphisms and Chronic Kidney Disease

In the last decade, GWAS have become essential for investigating the genetic contribution to CKD, with over 50 germline genetic loci identified as biomarkers of kidney disease risk or associated with SCr, cystatin-C and/or microalbuminuria (Cañadas-Garre et al., 2018a). The *UMOD* gene, coding for uromodulin, the most abundant urinary protein (Devuyst et al., 2017), is the gene with most of the consistently replicated genetic associations (Cañadas-Garre et al., 2018a). Several common *UMOD* variants (rs12917707, rs4293393, rs11864909, rs13329952) are associated with both CKD and eGFR (Köttgen et al., 2009, 2010; Gudbjartsson et al., 2010; Pattaro et al., 2012, 2016). More recently, the higher frequency in ESRD of another common *UMOD* variant (rs13333226), has been confirmed in 638 Chinese patients with ESRD and 366 controls (Chen et al., 2016). Several common variants in the myosin heavy chain type II isoform A (*MYH9*) gene have been associated with non-diabetic ESRD in African Americans (Kao et al., 2008; Kopp et al., 2008; Chambers et al., 2010). Common variants in *APOL1* are also associated with non-diabetic ESRD (Genovese et al., 2010; Tzur et al., 2010; Foster et al., 2013). Common variants in *ELMO1* gene have been associated with DKD and its progression to ESRD in several populations, although in this case with less consistency (Shimazaki et al., 2005; Leak et al., 2009; Pezzolesi et al., 2009a,b; Narres et al., 2016). A more recent meta-analysis of GWAS, including data from 133,413 individuals and subsequently replicated in 42,166 individuals, identified 24 new loci associated with eGFR (*BCAS1, AP5B1, A1CF, PTPRO, UNCX, NFKB1, TP53INP2, KCNQ1, CACNA1S, WNT7A, TSPAN9, IGFBP5, KBTBD2, RNF32, SYPL2, SDCCAG8, ETV5, DPEP1, LRP2, SIPA1L3, INHBC, ZNF204, SKIL*, and *NFATC1*) (Pattaro et al., 2016). The *trans*-ethnic meta-analysis showed that 12 loci had fully consistent effect direction on eGFR across European, Asian and African individuals (*SDCCAG8, LRP2, IGFBP5, SKIL, UNCX, KBTBD2, A1CF, KCNQ1, AP5B1, PTPRO, TP53INP2*, and *BCAS1*). Regarding other measures of kidney function, a variant rs1801239 in the *CUBN* gene was proposed as a predictor of UACR and microalbuminuria in a meta-analysis of 63,153 individuals of European ancestry (Böger et al., 2011), and another variant in the same gene, rs10795433, has been associated with UACR in 5,825 individuals of European ancestry with diabetes compared to 46,061 without diabetes (Teumer et al., 2016). A recent discovery GWAS of UACR in 382,500 unrelated European participants of the UK Biobank, a population-based cohort, reported 33 common variants, 20 of them sharing a consistent direction of effect with the study by Teumer et al. (2016), including *CUBN, HOTTIP, LOC101927609, NR3C2, ARL15, SHROOM3, MAPKBP1, ICA1L, SNX17, LRMDA, SBF2, SPATA5L1, FUT1/IZUMO1* genes and additional variants in chromosomes 1, 2, 7, 14, and 15: rs10157710, rs12032996, rs1276720, rs17158386, rs2023844, rs2472297, rs4410790, rs6535594, rs702634, rs7654754, rs8035855, rs10207567, rs1047891, rs4665972, rs13394343, rs67339103, rs17368443, rs4288924, rs1145074, and rs838142 (Haas et al., 2018). This GWAS also identified 11 common novel associations in *CUBN*, PRKCI, *EFNA3-EFNA1, MIR548AR-LOC646736, COL4A4, SPHKAP-PID1, INC01262-FRG1, RIB1-LINC00861*, and *BAHCC1* genes. UACR had previously been associated with another common variant in *SHROOM3* (rs17319721) in a meta-analysis of 31,580 and 27,746 Caucasian patients, although it did not reach GWA significance (*p*_discovery_ = 1.9 × 10^-6^) (Böger et al., 2011).

Although GWAS have successfully identified SNPs associations for the different traits associated with CKD, most of them are common DNA variants of small effect size. The proportion of phenotypic variance of eGFR explained by the 24 novel loci and the 29 previously identified by Pattaro et al. was 3.22%, therefore of limited help in CKD prediction (Pattaro et al., 2016).

An alternative to the concept of SNPs as single biomarkers is the use of PGRS, which provide individual estimates of the risk of presenting a determinate trait calculated from the combination of specific risks associated to SNPs. However, PGRS may provide only a partial solution in complex diseases. A recent analysis of 32 highly relevant traits related to five disease areas in 13,436 subjects of the Lifelines Cohort reported only 10.7% of the common-SNP heritability of these traits was explained by the different weighted PGRS, compiled from genome-wide significantly associated index SNPs based on previous GWAS (Nolte et al., 2017). The percentage of variance explained by the PGRS for SCr, composed of three SNPs of high imputation quality (*R*^2^ > 0.5) was 0.2% for both weighted and unweighted PGRS (Nolte et al., 2017). Addition of one low-quality SNP increased the variance up to 0.21% (weighted PGRS). For the eGFR PGRS, composed of 33 SNPs (high-quality), the percentage of variance was 1.6% for unweighted PGRS and 1.8% for the weighted PGRS. Addition of 19 low-quality SNPs increased the variance up to 2.01% (weighted PGRS). There were no high-quality SNPs associated with UACR, so it was not possible to construct this PGRS. The inclusion of one low-quality SNP explained 0.12% of the variance with both weighted and unweighted PGRS (Nolte et al., 2017). The PGRS for urate, composed of 20 SNPs (high-quality), explained from 2.0 to 4.2%, depending if either an unweighted or weighted PGRS was considered. Addition of eight low-quality SNPs increased the variance up to 4.52% (weighted PGRS) (Nolte et al., 2017).

Next generation sequencing has an increasing role for both research and diagnosis of kidney disease. Recently, a NGS panel for a spectrum of genetic nephropathies, covering 301 genes, was designed and validated in a CLIA-approved laboratory (Larsen et al., 2016). The assay showed excellent performance characteristics and was able to provide a specific molecular pathogenesis-based diagnosis in 46% of biopsies studied. An NGS panel covering all coding and regulatory regions of *UMOD* identified 119 genetic variants in 23 ESRD patients (compared to 22 controls without renal disease). Ninety of those variants were SNPs, 60 of them with minor allele frequency greater than 5%. Linkage disequilibrium allowed 20 SNPs to capture 100% of the alleles with a mean *R*^2^ of 0.97, providing a set of independent SNPs suitable for association analysis in larger cohorts (Bailie et al., 2017).

Whole-exome sequencing provided a diagnosis in 22 out of 92 adults with CKD of unknown cause, familial nephropathy or hypertension (22/92; 24%) (Lata et al., 2018). The confirmation of the clinical diagnosis by WES allowed the appropriate genetic counseling and screening for the family members of some affected patients and helped in clarifying or entirely reclassified the disease in other cases (Lata et al., 2018). WES also identified *PARN* haploinsufficiency as a new genetic cause of CKD in this study (Lata et al., 2018). The *PARN* gene encodes a poly(A)-specific ribonuclease which mediates the post-transcriptional maturation of the telomerase RNA component (*TERC)* and causes telomere disorders (Moon et al., 2015). Exome sequencing has recently identified 11 loci (*p* < 1 × 10^-4^) in eight genes (*PLEKHN1, NADK, RAD51AP2, RREB1, PEX6, GRM8, PRX, APOL1*) associated with T2DM-ESRD in 2476 cases and 2057 non-nephropathy control individuals of African American origin (Guan et al., 2018). However, exome data from 7974 self-identified healthy adults has recently demonstrated an implausibly high rate of candidate pathogenic variants for kidney and genitourinary diseases (1.4%), much higher than the prevalence of genetic renal/genitourinary disorders, even after stringent filtering criteria (removal of indels and minor allele frequency cutoffs of < 0.01% and <0.1% for dominant and recessive disorders, respectively) (Rasouly et al., 2018). This overestimation of potential pathogenic variants may increase the burden of uncertain diagnoses and medical referrals rather than alleviate it, therefore minimizing the utility of exome sequencing in clinical practice (Rasouly et al., 2018).

### Mitochondria and Their Association With Chronic Kidney Disease

Mitochondria are organelles which generate ATP through OXPHOS and thus represent the primary energy source for normal function of the cell and body (Cooper, 2000; Lodish et al., 2012; Chaban et al., 2014). The majority of mitochondrial proteins are encoded by nuclear genes (Timmis et al., 2004; Dolezal et al., 2006). However, mitochondria also have their own circular genome 16,569 base pairs long that contains 37 genes which encode 13 proteins of the electron transport chain essential for OXPHOS (Meiklejohn et al., 2013) along with two rRNAs and 22 tRNAs (Taanman, 1999; Cooper, 2000; Gray et al., 2008). Mitochondrial dysfunction in kidney tissue may severely impact renal health and has previously been implicated in CKD development (Rahman and Hall, 2013; Wallace, 2013; Zhan et al., 2013; Che et al., 2014; Douglas et al., 2014; Swan et al., 2015; Galvan et al., 2017).

**FIGURE 2:**
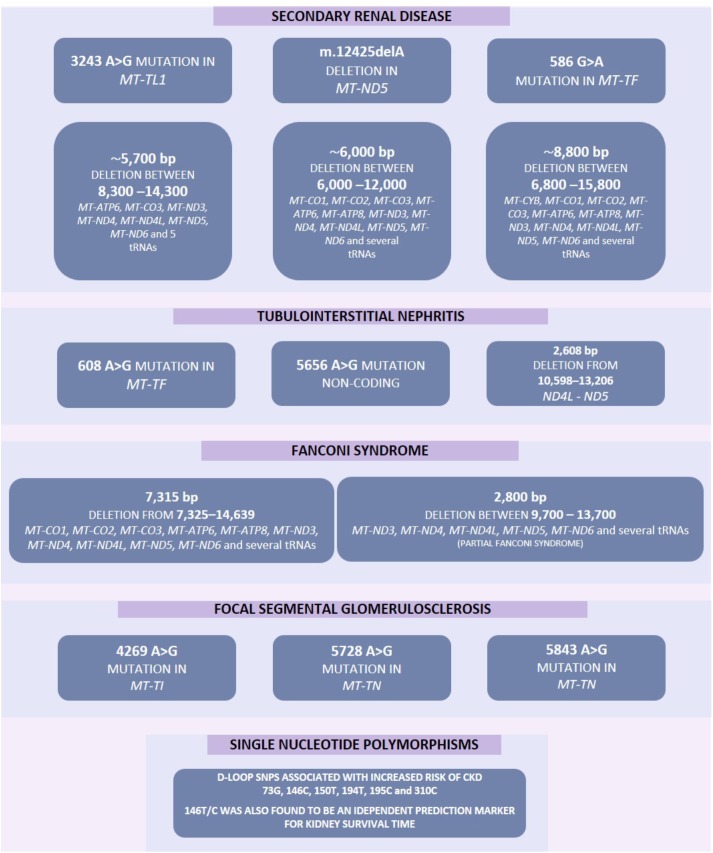
Increased mutation rate in mtDNA have previously been associated with several diseases including various forms of kidney disease. These mutations include point mutations, deletions and single nucleotide polymorphisms. Some of these mutations may result in several pathological phenotypes and these have been highlighted by a solid line between such genes.

If mitochondrial metabolism is adversely affected by genetic variants it can result in kidney disease, sometimes as part of a wider clinical disorder (Rahman and Hall, 2013). Somatic mtDNA mutations may be associated with aging, resulting in decline of mitochondrial function in older individuals (Wallace, 2013). Increased levels of mtDNA mutations have previously been associated with several disorders including various forms of kidney disease (Figure 2) (Wallace, 2013).

**FIGURE 3:**
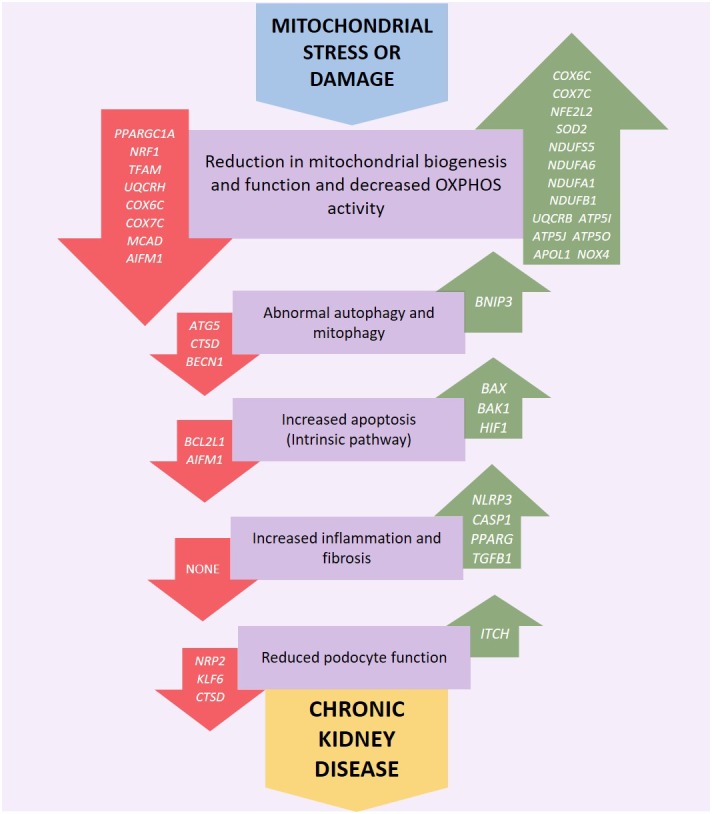
Genetic variation or altered expression of nuclear genes which encode mitochondrial proteins may impair respiratory chain complex activities leading to an increase in production of reactive oxygen species (ROS). This initiates a negative feedback loop, further reducing mitochondrial function, and ATP production along with an increase in OXPHOS defects and ROS generation leading to increased oxidative stress which may lead to uncontrolled autophagy, mitophagy and further ROS production. Mitochondrial dysfunction, ROS generation and the resulting dysregulation of autophagic mechanisms may also activate intrinsic apoptotic mechanisms resulting in inflammation and fibrosis in the renal tubules, glomerulus and podocytes eventually leading to kidney disease. Red arrows indicate underexpression and green arrows overexpression.

Mitochondrial dysfunction can occur via a number of pathways, for example persistent hyperglycemia (associated with diabetes) results in increased tubular oxygen consumption, and in turn leads to hypoxia of the kidney tissue (Hansell et al., 2013). Mitochondrial dysfunction can be associated with increased electron leakage from the respiratory chain during OXPHOS, which results in ROS being generated which can cause kidney injury (Granata et al., 2015) including direct damage to DNA (Marnett, 2000). Genetic variation in mtDNA (Figure 2) or nuclear genes (Figure 3) which influence mitochondrial function may impair respiratory chain complex activities leading to an increase in production of ROS resulting in a negative feedback loop, increasing mitochondrial dysfunction, OXPHOS defects and ROS generation along with a reduction in ATP production which leads to increased oxidative stress which may lead to uncontrolled autophagy, mitophagy, and further ROS production (Fernandez-Marcos and Auwerx, 2011; Kim et al., 2012; Zaza et al., 2013). Mitochondrial dysfunction, ROS generation and the resulting dysregulation of autophagic mechanisms may also lead to an upregulation of the intrinsic pathway of apoptosis which in turn leads to inflammation and fibrosis in the renal tubules and glomeruli (Tanaka et al., 2005; Song et al., 2010; Ye et al., 2010; Coughlan et al., 2016).

Despite the mitochondrial genome being widely ignored in relation to CKD, a number of studies have identified mitochondrial genomic loci associated with specific forms of renal disease (Table 1). SNPs within *MT-HV2, MT-CO1*, and *MT-CO2c* have been associated with IgAN (Douglas et al., 2014); the A3243G point mutation in the leucine^UUR^ tRNA gene (*MT-TL1*) was identified in patients with FSGS (Jansen et al., 1997; Kurogouchi et al., 1998; Nakamura et al., 1999; Doleris et al., 2000; Hotta et al., 2001; Hirano et al., 2002; Guéry et al., 2003), other forms of renal disease (Guéry et al., 2003) and in a male with a history of MELAS syndrome including kidney cancer, who rapidly developed renal failure after removal of the cancerous kidney (Piccoli et al., 2012). In general, mtDNA biomarkers have not been considered as potential biomarkers in association studies, therefore most findings concerning the mitochondrial genome in relation to CKD come from case reports. The *MT-TW* tRNA (m.5538 G > A) mutation was identified as causing FSGS in a male (Lim et al., 2017). The (m.547 A > T) and tRNA^Phe^ (m.616 T > C) mutations were found in patients suffering from inherited tubulointerstitial kidney disease, who did not display typical symptoms of mitochondrial disease (Connor et al., 2017). A novel mutation in mtDNA (09155 A > G) was described in a Caucasian female with a history of renal disease, and symptoms of Maternally inherited deafness and diabetes (MIDD) (Adema et al., 2016). Mutations in nuclear genes associated with mitochondrial function have also been associated with renal disease. The P99L mutation in the *BCS1L* gene was found in a female infant suffering from Neonatal Toni–Debré–Fanconi Syndrome, including renal tubulopathy (Ezgu et al., 2013). R45C and R56X mutations in the *BCS1L* gene were described in two siblings suffering from congenital lactic acidosis, including renal tubulopathy (De Meirleir et al., 2003), and nine different mutations in *FBXL4* were identified in nine individuals suffering from mitochondrial encephalomyopathy including renal tubular acidosis (Gai et al., 2013).

**Table 1:** Studies in mitochondrial genome and in nuclear genes associated with mitochondrial function (FSGS, focal segmental glomerulosclerosis; MELAS, mitochondrial encephalomyopathy, lactic acidosis, and stroke-like episodes; MIDD, maternally inherited diabetes and deafness).

Gene	Mutation	Disease	Methodology	Function	References
?	09155 A > G	MIDD, renal disease	Whole exome sequencing	?	Adema et al., 2016
MT-TL1	3243 A > G	MELAS syndrome, renal cancer, renal failure	Restriction fragment length polymorphism (ApaI)	Encodes tRNALeu(UUR), forms amino acids containing Leucine	Piccoli et al., 2012
		“Kidney disease”	Allele-specific PCR	Encodes tRNALeu(UUR), forms amino acids containing Leucine	Guéry et al., 2003
		FSGS	Restriction fragment length polymorphism (ApaI)	Encodes tRNALeu(UUR), forms amino acids containing Leucine	Kurogouchi et al., 1998
		Kidney disease, diabetes, hearing loss	Restriction fragment length polymorphism (ApaI)	Encodes tRNALeu(UUR), forms amino acids containing Leucine	Jansen et al., 1997
		Kidney disease, diabetes, hearing loss, cataract	Restriction fragment length polymorphism (ApaI)	Encodes tRNALeu(UUR), forms amino acids containing Leucine	Nakamura et al., 1999
		FSGS, diabetes, hearing loss (3/4 cases), Cerebellar syndrome (1/4 cases)	Allele-specific amplification	Encodes tRNALeu(UUR), forms amino acids containing Leucine	Doleris et al., 2000
		FSGS, diabetes (2/4 cases), hearing loss (1/4 cases)	Restriction fragment length polymorphism (ApaI)	Encodes tRNALeu(UUR), forms amino acids containing Leucine	Hotta et al., 2001
		Renal disease, hearing loss	?	Encodes tRNALeu(UUR), forms amino acids containing Leucine	Hirano et al., 2002
Control region	m.547 A > T	Inherited tubulointerstitial kidney disease	Sanger sequencing	?	Connor et al., 2017
MT-TF	m.616 T > C	Inherited tubulointerstitial kidney disease	Sanger sequencing	Encodes tRNA Phenylalanine, forms amino acids containing phenylalanine	Connor et al., 2017
MT-TW	m.5538 G > A	FSGS	Whole exome sequencing	Encodes tRNA tryptophan, forms amino acids containing tryptophan	Lim et al., 2017
BCS1L	P99L	Neonatal Toni–Debré–Fanconi Syndrome, renal tubulopathy	?	Assembly of mitochondrial respiratory chain complex III	Ezgu et al., 2013
BCS1L	R45C, R56X	Congenital lactic acidosis, renal tubulopathy	Direct sequencing	Assembly of mitochondrial respiratory chain complex III	De Meirleir et al., 2003
FBXL4	(c.1703G > C [p.Gly568Ala], c.1444C > T [p.Arg482Trp], c.1694A > G (p.Asp565Gly), c.1652T > A (p.Ile551Asn), c.1067del (p.Gly356Alafs^∗^15), c.1790A > C (p.Gln597Pro), (c.[614T > C; 106A > T], p.[Ile205Thr; Arg36^∗^]), (c.1229C > T [p.Ser410Phe])	Mitochondrial encephalomyopathy, renal tubular acidosis	Whole exome sequencing	Phosphorylation-dependent ubiquitination in mitochondria	Gai et al., 2013


Despite the limited published literature, the known significance of the mitochondrial genome with relation to renal function and the multiple case reports relating to individuals suffering from renal dysfunction associated with mutations in mitochondrial or mitochondrial-associated genes, suggest that there exists considerable potential for genetic mutations, resulting in mitochondrial dysfunction, to contribute toward CKD.

### X and Y Chromosomes

In CKD research, despite the efforts of extensive GWAS and other genomic analyses in this area, a “blind spot” still exists in the form of X- and Y-chromosome analysis. Fifty-three of the 3,643 publications found in the online GWAS catalog (hosted by the National Human Genome Research Institute-European Bioinformatics Institute) examined CKD and/or kidney-associated traits (MacArthur et al., 2017). Over 450 genome-wide associations (*p* < 5 × 10^-8^) with renal disease and/or related traits were found at 140 loci across the genome (Table 2).

**Table 2:** Comparison of associations reaching genome-wide significance (5 × 10^-8^) per chromosome in renal disease or related traits (bp, base pairs; Chr, chromosome; GWAS, genome-wide association studies).

Chr	Size Chr (bp)	Coding Genes per Chr	Number of Associations with Renal Traits in GWAS Catalog	
			
			Total	Renal-Associated Loci
1	248956422	2050	21	15
2	242193529	1301	47	12
3	198295559	1079	18	8
4	190214555	753	25	6
5	181538259	884	19	7
6	170805979	1045	60	11
7	159345973	992	26	6
8	145138636	1021	16	5
9	138394717	778	8	3
10	133797422	731	14	6
11	135086622	1316	33	10
12	133275309	1036	26	10
13	114364328	321	7	3
14	107043718	821	1	1
15	101991189	616	33	8
16	90338345	862	29	5
17	83257441	1188	24	4
18	80373285	269	13	5
19	58617616	1474	9	4
20	64444167	543	9	5
21	46709983	232	2	1
22	50818468	492	8	3
X	156040895	846	4	2
Y	57227415	63	0	0
			Total: 452	Total: 140


As depicted in Table 2, the number of associations per chromosome is the lowest for chromosome Y (no associations) and the fourth lowest number of associations for chromosome X (four associations). This is not surprising for chromosome Y. Historically thought of as a “genetic wasteland” (Skaletsky et al., 2003), association analyses usually exclude the Y-chromosome. Indeed, in the 53 studies examining renal disease/traits, only one included the Y-chromosome in the association analysis (Nanayakkara et al., 2014). Given that the Y-chromosome is the smallest and contains the fewest number of genes per number of base pairs (Zerbino et al., 2018), the lack of significant associations in this study is not unexpected.

However, for a chromosome of its size and gene content, the small number of associations found between X-chromosome SNPs and renal disease/traits raises questions as to why there are so few reported. Indeed, the only chromosomes with fewer reported associations are chromosomes 14 and 21, both of which are smaller and contain fewer genes than chromosome X. The lack of reported associations with sex chromosome SNPs could be due to a true lack of association or under-representation of sex chromosomes in GWAS.

Of the 53 GWAS in renal disease/traits, 10 are unclear as to whether X- and Y-chromosome SNPs were included in association analysis. Over half (62%) of the studies did not report sex chromosome association results, with many actively excluding the X- and Y-chromosomes from the association (Chambers et al., 2010; McDonough et al., 2011) or meta-analysis stages (Köttgen et al., 2009). Of the 10 studies (18%) that explicitly state that the X-chromosome analysis was included, only one study found associations between X-chromosome SNPs and renal traits (SCr and eGFR) that reached genome-wide significance (Kanai et al., 2018). Two SNPS, rs12845465, and rs5987107, were both associated with SCr and eGFR (*p* < 5 × 10^-8^). In only one study does Y-chromosome analysis appear to be included, where no SNPs reached genome-wide significance (Nanayakkara et al., 2014).

Therefore, with less than 20% of studies reporting X-chromosome results and Y-chromosome exclusion almost ubiquitous, it is not surprising that very few sex chromosome SNPs have shown association in studies of renal disease/traits. A possible explanation for sex chromosome exclusion is that traditional imputation methods call for the use of autosomes only (Marchini et al., 2007). Even now that methods of X-chromosome imputation have been introduced (Marchini and Howie, 2010; König et al., 2014), greater expertise is required and the X-chromosome is imputed separately from the autosomes, and these issues may lead some researchers to simply exclude it. The lack of reported analysis of X-chromosome SNPs in renal disease then leads to its exclusion from meta-analysis, as X-chromosome results are not common between all included studies. Poor genotyping of X-chromosome SNPs may also account for a reduced number of significant associations. Evidence has suggested that removal of X-chromosome SNPs during quality control is significantly more likely, due to a higher rate of chromosomal anomalies or missing call rate than autosomal SNPs (Wise et al., 2013). However, despite the successful imputation of the X-chromosome, chromosome Y lags behind. Despite recent efforts (Zhang et al., 2013), haplogroup-based Y-chromosome imputation is still not widely used, with authors opting to instead use only directly genotyped SNPs (Charchar et al., 2012).

The lack of sex chromosome inclusion in CKD GWAS may be one reason that the relationship between sex and CKD incidence/progression is so unclear. By regularly excluding these chromosomes from renal GWAS, we may miss SNPs that infer either increased CKD risk or protection to one gender in particular.

Traditionally, a greater risk of CKD incidence and progression to ESRD was associated with males. While current evidence still supports an increased rate of progression in men to ESRD (Yang et al., 2014), the risk inferred by gender on incidence of CKD is unclear. A study which used several definitions of incidence found that when using eGFR-based definitions of CKD (<60 ml/min/1.73^2^), incident CKD was significantly higher in women than men (*p* = 0.02), but when using a minimum increase in SCr to detect CKD, men had a significantly higher incidence (*p* = 0.001) (Bash et al., 2009). Gender adjustment occurs in eGFR calculation, which may explain this difference. A study conducted to develop a CKD risk score also found that female sex was associated with prevalent CKD (*p* = 0.02) (Bang et al., 2007), as did a Turkish population study (*p* < 0.001) (Süleymanlar et al., 2011). Additionally, a comprehensive review revealed that 38 studies found CKD was more prevalent in women, while 13 found it was more prevalent in men (Hill et al., 2016). Therefore, while women seem to make up a larger proportion of the individuals affected by CKD, affected men seem to progress at a much faster rate, highlighting the difference in the way that CKD affects men and women.

Clinical evidence and recent literature support a link between the sex chromosomes and impaired renal function. Arising as a result of a mutation in *COL4A5* on chromosome X, Alport syndrome is caused by impaired production or function of collagen in various basement membranes throughout the body, including in the glomerulus (Kashtan, 2017). The condition is characterized by hearing loss, ocular abnormalities and progression to ESRD, where up to 30% of women reach ESRD by age 60 (Savige et al., 2016) and the majority of affected males will require transplant or dialysis by their late twenties (Temme et al., 2012).

## Discussion and Conclusion

Extensive efforts have been made to harness existing GWAS data and improve the sample size statistical power via GWAS meta-analyses to uncover true associations between genetic variants and CKD. Nevertheless, it remains challenging to explain all of the heritability of CKD with currently available methods and datasets.

The definition of CKD phenotype (based on SCr, eGFR and/or urinary albumin measurements) varies between published studies which impacts on the strength of genetic associations observed. CKD is phenotypically heterogeneous and CKD risk may be amplified by co-morbidities such as obesity. Many genetic studies have a cross-sectional case-control design with the determination of CKD based on a single measurements of kidney function. This limits the ability to explore dynamic gene-environment interactions over time, e.g., the impact of diet, gut microbiome, smoking, physical activity, stress, medication use or long-term glycemic control on genetic risk of developing CKD (Simon et al., 2016; Sandoval-Motta et al., 2017).

Prospective follow up of longitudinal cohorts at risk of developing CKD, such as the UK Biobank population may help to unravel some of the complex interplay of genetic background and environmental stressors contributing to kidney damage (Kim et al., 2017). Stratification by co-morbidity, e.g., elevated BMI in T2DM patients, may help identify additional risk variants with a stronger genetic predisposition to CKD (Perry et al., 2012).

The molecular biomarkers for CKD that have received less attention (telomeres, CNVs, mtDNA variants, X and Y chromosomes) are pieces of the missing heritability puzzle. Shorter telomere length is associated with renal dysfunction and CKD progression, even though reported results are conflicting. CNVs have been linked to CAKUT (1q23.1, 22q11, 4p16.1, 7q33, 8q13.2q13.3, and 17q12 regions), PUV (3p25.1p25.2 and 17p12), nephronophthisis (*NPHP1* gene) and IgAN (*DEFA1A3* locus). Information on mtDNA biomarkers is mostly from case reports, but the A3243G mutation in the *MT-TL1* gene has been associated with FSGS. One GWAS has found associations between X-chromosome SNPs and renal function (rs12845465 and rs5987107). No SNPs in the Y-chromosome have reached genome-wide significance.

Unraveling the missing heritability of CKD will need coherent integration of the different sources contributing to total heritability, and not just inclusion of missing gene variants. Using multiple –“omics” data by combining elements of the phenome, genome, epigenome, transcriptome, metabolome, proteome, and microbiome and translating these data into a useful individual CKD risk assessment remains a major challenge. These research goals efforts will likely help to increase our understanding of the mechanisms of kidney function and disease, and improve disease prediction.

## Author Contributions

MC-G, KA, RC, RS, LJS, AJM, and APM contributed to the conception or design of the work, acquisition, analysis and interpretation of data for the work, drafting the work and revising it critically for important intellectual content, provided the approval for publication of the content, and agreed to be accountable for all aspects of the work in ensuring that questions related to the accuracy or integrity of any part of the work are appropriately investigated and resolved.

## Conflict of Interest Statement

The authors declare that the research was conducted in the absence of any commercial or financial relationships that could be construed as a potential conflict of interest.

## Abbreviations

ATP: adenosine triphosphate
CAKUT: congenital anomalies of the kidney and urinary tract
CKD: chronic kidney disease
CKiD: chronic kidney disease in children cohort study
CNVs: copy number variants
CRISIS: chronic renal insufficiency standards implementation
DKD: diabetic kidney disease
eGFR: estimated glomerular filtration rate
ESRD: end-stage renal disease
FSGS: focal segmental glomerulosclerosis
GWAS: genome-wide association studies
IgAN: immunoglobulin A nephropathy
MMKD: mild to moderate kidney disease
mtDNA: mitochondrial DNA
NGS: next generation sequencing
NPH: nephronophthisis
OXPHOS: oxidative phosphorylation
PGRS: polygenic risk scores
PREVEND: Prevention of Renal and Vascular Endstage Disease study
PUV: posterior urethral valves
ROS: reactive oxygen species
SCr: serum creatinine
SNVs: single nucleotide variants
T2DM: type 2 diabetes mellitus
UACR: urinary albumin/creatinine ratio
WES: whole-exome sequencing
